# Phyllosphere of *Agathis australis* Leaves and the Impact of the Soil-Borne Pathogen *Phytophthora agathidicida*

**DOI:** 10.1007/s00248-024-02441-9

**Published:** 2024-10-09

**Authors:** Maisie Leigh Hamilton Murray, Andrew Dopheide, Jenny Leonard, Mahajabeen Padamsee, Luitgard Schwendenmann

**Affiliations:** 1 School of Biological Sciences, University of Auckland, 3A Symonds Street, Auckland, 1010 New Zealand; 2 Manaaki Whenua—Landcare Research, 231 Morrin Road St Johns, Auckland, 1072 New Zealand; 3 School of Environment, University of Auckland, 23 Symonds Street, Auckland, 1010 New Zealand

**Keywords:** Fungi, Kauri, Leaf leachate, Microbiota, Oomycota, Phylloplane

## Abstract

Leaf surface microbial communities play an important role in forest ecosystems and are known to be affected by environmental and host conditions, including diseases impacting the host. *Phytophthora agathidicida* is a soil-borne pathogen that causes severe disease (kauri dieback) in one of New Zealand’s endemic trees, *Agathis australis* (kauri). This research characterised the microbial communities of the *A. australis* phyllosphere (i.e. leaf surface) using modern molecular techniques and explored the effects of *P. agathidicida* on those communities. Fresh leaves were collected from trees where *P. agathidicida* was and was not detected in the soil and characterisation of the leaf surface microbial community was carried out via high-throughput amplicon sequencing of the internal transcribed spacer (ITS) and 16S ribosomal RNA regions. Nutrients in leaf leachates were also measured to identify other possible drivers of microbial diversity. The dominant phyllosphere microbial phylum was Proteobacteria followed by Acidobacteria. The phyllosphere microbial richness of *A. agathis* associated with *P. agathidicida-*infected soils was found to be generally lower than where the pathogen was not detected for both prokaryote (bacterial) and fungal phyla. Leaf leachate pH as well as boron and silicon had significant associations with bacterial and fungal community structure. These findings contribute to the development of a comprehensive understanding of *A. australis* leaf surface microbial communities and the effects of the soil pathogen *P. agathidicida* on those communities.

**Supplementary Information:**

The online version contains supplementary material available at 10.1007/s00248-024-02441-9.

## Introduction

Microbiota colonise both internal and external leaf tissues. Those of the leaf surface can include a variety of bacteria, yeasts, protozoa, and fungi, and the composition of these communities is responsive to both host and environmental conditions [1]. In cases where the host experiences abiotic and biotic stress, it might be expected that the leaf-associated microbial community and the communities of the wider phyllosphere (i.e. the total above-ground surface of the plant) will also be impacted [2, 3].

One factor that may impact the host, and therefore the microbiota of leaves, is the presence of pathogens. Past research has investigated the impacts of foliar pathogens on phyllosphere microbial communities associated with infected plants [4–7]. Lower microbial diversity in or on leaves and/or twigs has been found for *Lophodermium*-diseased needles of Scots pine (*Pinus sylvestrus*) [4], unhealthy/yellow *Citrus limon* foliage infected with *Colletotrichum gloeosporioides* or *Mycosphaerella* pathogenic species [5], and the twigs of three cultivars of olive (*Olea europaea*) symptomatic of olive knot disease (*Pseudomonas savastoni* infection) [6]. Gomes et al. [6] identified a decline in abundance, richness, and diversity of fungal groups associated with symptomatic *Olea europaea* trees, with some fungal families disappearing altogether under disease conditions.

Microbial communities of the phyllosphere can also be affected by pathogens that impact the plant’s roots. For example, root infection of chestnut (*Castanea satvia*) by *Phytophthora cinnamomi* changed the physical and chemical characteristics of the leaves, reduced microbial colonisation, and inhibited microbial activity [7]. Another study [8] found that phyllosphere and rhizosphere communities are altered by biotic stress caused by root pathogens. These are among the handful of studies that have assessed differences in foliar microbial communities between plants with healthy and diseased roots.

*Agathis australis* is a member of the ancient coniferous Araucariaceae family and the southernmost species of the *Agathis* genus [9]. *Agathis australis* is one of the longest-lived and largest tree species endemic to New Zealand [10] and has been described as a foundation species with regard to its ability to affect environmental conditions, ecosystem processes, and species composition underneath its canopy [11]. The leaves of this tree are long lived, remaining on twigs for up to 15 years [12]. *Agathis australis* also has great cultural and spiritual significance for many iwi (Māori tribes) [13] and is known to many as a ‘rākau rangatira’ (chiefly tree).

The survival of *A. australis* is threatened by kauri dieback caused by the soil/water-borne Oomycota *Phytophthora agathidicida* [14]. There is no cure for *P. agathidicida* infection, and once infected, tree death is inevitable [15]. As *A. australis* play important ecological and cultural roles, numerous studies have been undertaken to understand the impact of *P. agathidicida* on *A. australis*-dominated forests. For example, Byers et al. [16] have demonstrated that soil microbial communities change due to the presence of the pathogen, and Schwendenmann et al. [17, 18] showed that canopy and forest floor nutrient and carbon fluxes decreased with increasing *P. agathidicida* infection.

A knowledge gap exists in relation to the composition and diversity of microbial communities of the *A. australis* phyllosphere. It is important to characterise these communities, not least because of the potential value of these organisms to the canopy ecosystem and the host directly, e.g. through disease-suppressive soil-microbiomes [19], but also because the species found on the living leaf may affect leaf litter characteristics and thereby influence litter decomposition and nutrient cycling through the forest ecosystem [20]. *Phytophthora agathidicida* infects the roots of *A. australis*, causing damage to the roots and vascular tissue and consequently impacting nutrient transport through the tree [14]. This leads to the formation of chlorotic leaves, defoliation, and canopy dieback [21]. Studies have demonstrated that infection by soil-borne *Phytophthora* species alters macro- and micronutrient concentrations in leaves (e.g. [22]). We assume that *P. agathidicida* infection affects the characteristics of leaves (e.g. nutrient content), which in turn may affect the phyllosphere microbial communities [16]. Our knowledge of microbial communities in the *A. australis* canopy is incomplete [23, 24], and the differences in phyllosphere microbial communities between trees with and without *P. agathidicida* in the surrounding soils have not been interrogated.

This project aimed to characterise the microbial communities on the surfaces of living *A. australis* leaves and compare differences in these communities under conditions where *P. agathidicida* was and was not detected in the soil. Leaf surface conditions (pH and nutrients in leaf leachates) were also measured to identify other possible drivers of microbial diversity.

## Methods

### Study Sites

Leaf samples were collected from *A. australis* trees at three locations (Cascades, Huia, and Piha) in the Waitākere Ranges Regional Park, Auckland, New Zealand (Supplemental Fig. 1). The Waitākere Ranges Regional Park comprises of regenerating podocarp-broadleaf forest growing on sandy loam soils (andesitic grit, sand, and siltstone) [25]. The mean annual temperatures in the area are from 13 to 16 °C, and the mean annual total rainfall, based on data from 1981 to 2010, is between 1300 and 1600 mm [26].

**Fig. 1:**
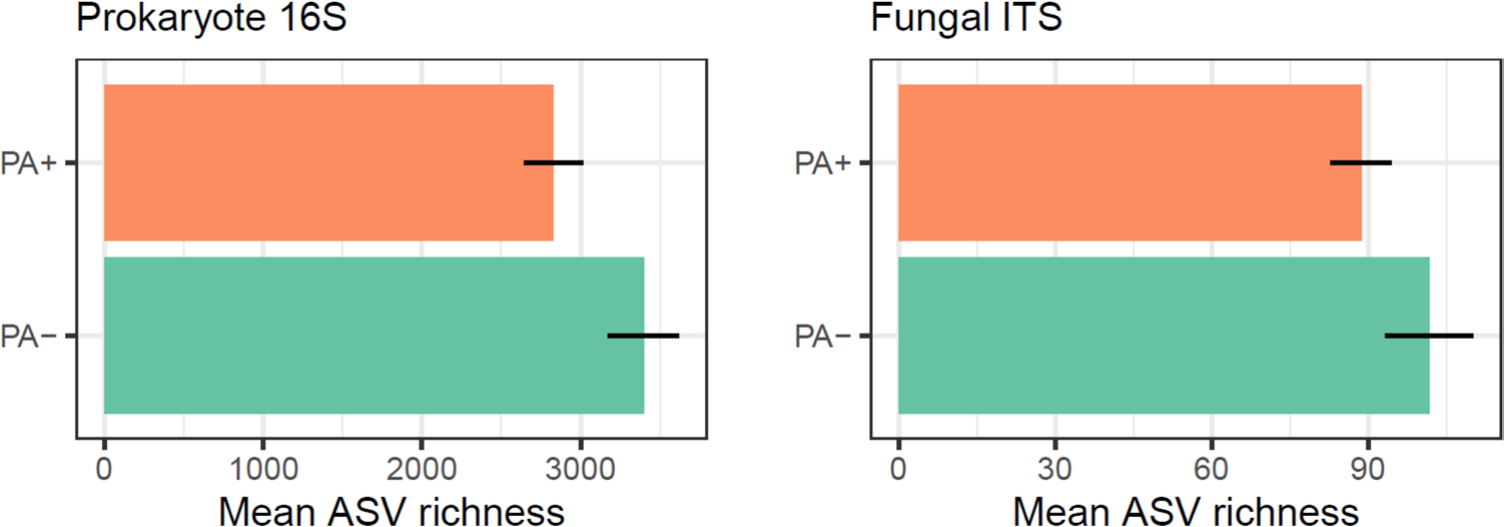
Mean richness (± SE) of prokaryotes and fungi found on the phyllosphere (i.e. leaf surface) of *A. australis*. Comparisons are between conditions where *P. agathidicida* was detected (PA +) or not detected (PA −) in the associated soil of the sampled tree

At each location, two 40 m × 50 m plots had been established for long-term vegetation monitoring between 2011 and 2014. The distance between the two plots at a given location ranged from 10 to 500 m. At each plot, there are four *A. australis* individuals, a minimum of 40 m apart, that are tagged for ongoing research. Tree heights were estimated at Cascades (14–35 m), Piha (9–17 m), and Huia (12–16 m).

For each pair of plots, one was originally characterised as ‘symptomatic’ and the other as ‘asymptomatic’ for kauri dieback disease. However, emerging evidence of misalignment between visual symptoms (which can be caused by other stressors, like drought) and the actual presence of *P. agathidicida* [27] prompted the decision to confirm pathogen presence using soil samples. Soil samples were collected from the base of each tree and placed in sealed bags on ice for transport to the lab. The soil samples were tested for the presence of *P. agathidicida* using loop-mediated isothermal amplification (LAMP) [28]. The pathogen was detected in the soils of 15 trees and was not detected in the soils of 9 trees (Supplemental Table 1). We grouped the trees by the pathogen condition (*P. agathidicida* detected/not detected) for our analyses and not by plot.

### Sampling Procedures

Sampling was undertaken over four consecutive days at the end of February 2022 (austral summer). Collection heights varied due to overall tree height and safe access. For each sampled tree (*n* = 4 per plot, 24 trees in total), one branch (~ 1 cm in diameter, ~ 50 cm in length) was taken from the upper (< 29.4 m height) and one from the lower (> 6.4 m in height) canopy (48 samples in total). The branches were placed in sealed plastic bags on ice for transport to the lab where they were immediately processed.

### Phyllosphere Microbiota and Leaf Leachates

To characterise phyllosphere microbiota, which for the purposes of this study focused on leaf surface microbiota, leaves from both older and more recent growth were selected from each sample. Leaves were only chosen if they were green in colour (i.e. not desiccated) and showed minimal damage to reduce the chance of unintentionally capturing internal leaf microbes in the wash. Depending on leaf size, between 10 and 25 leaves were collected across three to five randomly chosen twigs per branch and placed in 50-mL falcon tubes for washing.

Using a modified protocol of Tian et al. [29], 30 mL of a sterile wash solution (0.9% w/v NaCl + 0.01% Tween 80) was added to each falcon tube, which was then placed in an ultrasonic bath (Sonorex Super RK 106, Bandelin, Berlin, Germany) for 15 min. Following sonication, the wash was separated from the leaves of up to three twigs (depending on twig size) and collected in a separate 50-mL falcon tube for immediate refrigeration. Microbes in the wash were pelleted by centrifuge at 8000 rpg, and DNA was extracted using a 96-well extraction kit for DNA from plants (NucleoSpin 96 Plant II, Machery-Nagel, Düren, Germany), according to the manufacturer’s instructions. The extracted material was then sent to Genewiz®, China (https://www.genewiz.com/), for 16S ribosomal RNA and ITS amplicon sequencing on the Illumina MiSeq 2 × 250 platform.

Another three to five twigs per branch were used for leaching. Whole twigs were placed in a plastic bag with 500 mL of deionised water. The bag was placed on a Ratek Platform Mixer for 10 min. Washes were collected in 500-mL sealed plastic bottles, and pH was analysed using an Ohaus Starter 3100 M pH meter. Each wash was then vacuum filtered using Kimax USA Vacuum Flasks and Sartorius Polyethersulfone membrane filters (0.45 µm). The filtered solutions were analysed for boron (B), calcium (Ca), potassium (K), magnesium (Mg), manganese (Mn), sodium (Na), silicon (Si), and zinc (Zn) on an Agilent 7700 ICP-MS in helium mode (He mode) to reduce polyatomic interferences, except for B, which was run in no gas mode. Calibration standards were prepared in a 2% HNO_3_ solution from 1000-ppm single element standards (CPI International, USA). A 20-ppb solution of yttrium was used to monitor drift and matrix effects. Dissolved organic C and total N concentrations were measured using a Shimadzu Total Organic Carbon (TOC-L) Combustion Analyzer with a TNM-1 module. Results are presented in parts per million (ppm).

### Bioinformatic Processing and Data Analyses

PCR primers and sequencing adaptors were removed using Cutadapt (v3.5) [30]. The trimmed sequences were then cleaned of duplicates, errors, and ambiguous bases; filtered for lengths; and then denoised into amplicon sequence variants (ASVs) using VSEARCH (v2.17.1) [31]. The trimmed sequences were then mapped to the ASVs at a 97% identity threshold to infer their abundance. Taxonomic identities were then assigned to the ASVs using the Ribosomal Database Project (RDP) naïve Bayesian classifier (v2.13) [32] with the included 16S rRNA database for prokaryote data and the UNITE ITS database [33] for fungal data.

All data visualisation and statistical testing were performed using R software, version 4.1.3, in R studio [34], including the packages dplyr [35], vegan [36] for biodiversity and multivariate analyses, and ggplot2 [37] for data visualisation.

Mean ASV richness was calculated for prokaryotes and fungi under the *P. agathidicida* detected and not detected conditions across all phyllosphere samples (48 in total), for the three sites (Cascades, Huia, and Piha), for overall communities, and for individual phyla represented by at least 100 ASVs across the entire datasets. Mean richness values were compared, between *P. agathidicida* detected and not detected conditions, for overall communities, and for each phylum with at least 100 ASVs, using linear mixed models calculated using the R package lmerTest [38], with *P. agathidicida* condition as a fixed factor, and sample nested within site as a random variable (for tests across all sites), or sample as a random variable (for tests within sites).

Bray–Curtis distances were calculated between the proportional abundances of bacterial and fungal ASVs per sample, after which non-metric multidimensional scaling ordinations were generated. Distance-based redundancy analyses were carried out to investigate associations between leaf leachate measurements and bacterial and fungal phyllosphere community structure, visualised as vectors overlaid upon multidimensional scaling ordinations.

## Results

### Phyllosphere Microbial Communities

A total of 13,718 prokaryote ASVs and 383 fungal ITS ASVs were detected across all samples; no significant differences were detected between communities in upper and lower canopy samples using a PERMANOVA. The prokaryote ASVs were dominated by Proteobacteria (8997 ASVs), followed by Acidobacteria (1515), Planctomycetes (852), Cyanobacteria (725), Bacteroidetes (480), Firmicutes (378), Actinobacteria (268), and 39 further phyla each represented by fewer than 100 ASVs. The fungal ITS ASVs were dominated by Ascomycota (189 ASVs), followed by Basidiomycota (98), unidentified fungi (93), two Chytridiomycota ASVs (both detected in all samples), and a single Monoblepharomycota ASV.

On average across the three sites, the richness of prokaryote 16S ASVs per tree sample was 3395 (± 225) where *P. agathidicida* was not detected, compared to 2830 (± 187) where the pathogen was detected (Fig. 1). The richness of fungal ITS ASVs showed a similar trend, with an average of 102 (± 8.4) ASVs per tree sample where the pathogen was not detected, compared to 89 (± 5.8) where the pathogen was detected (Fig. 1). The prokaryote richness difference was supported by statistical evidence (*t*_44.5_ = 2.731, *p* = 0.009), but the fungal richness difference was not (*p* > 0.05).

**Fig. 2:**
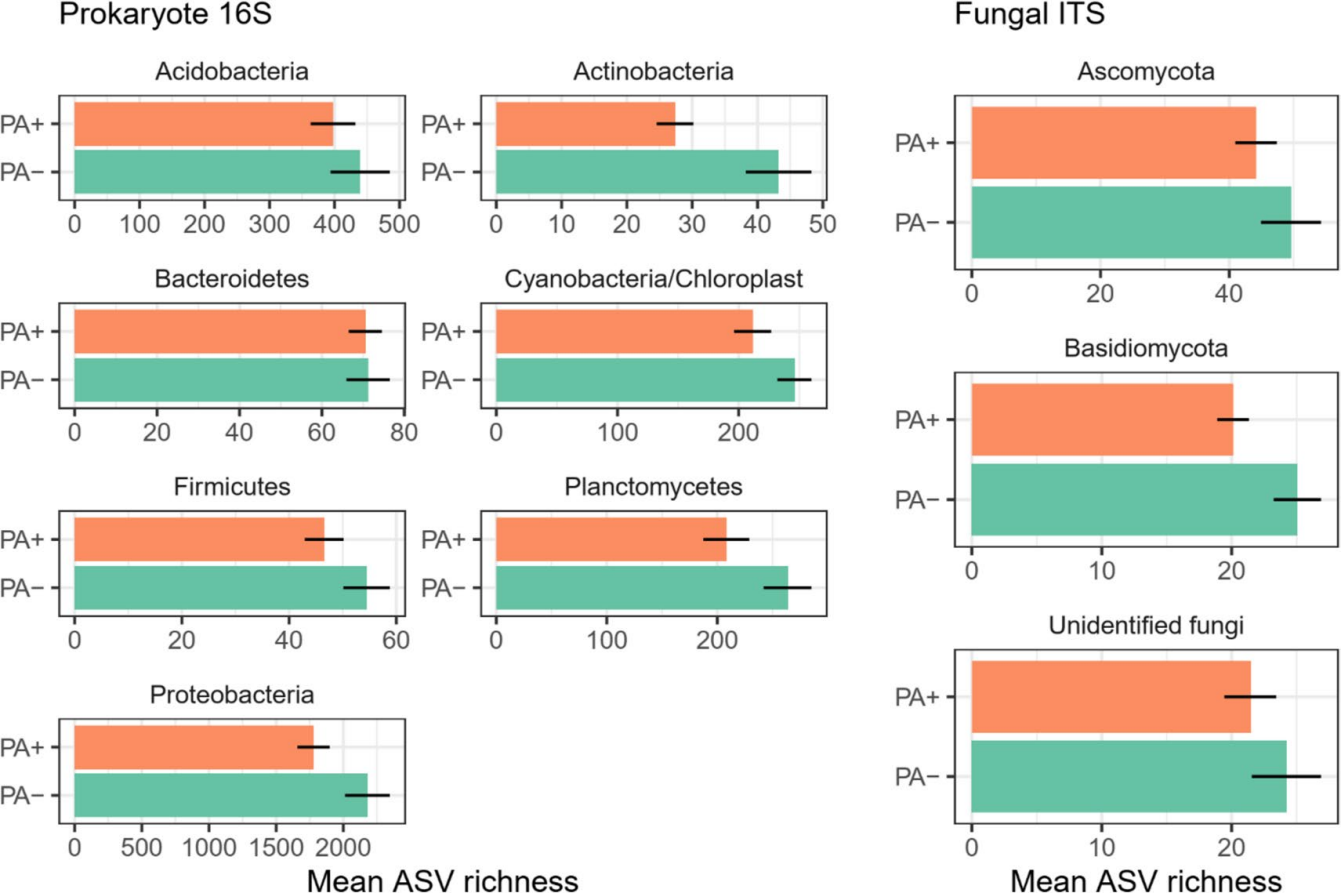
Mean ASV richness (± SE) of prokaryote and fungal phyla on the phyllosphere (i.e. leaf surface) of *A. australis*. Comparisons are between conditions where *P. agathidicida* was detected (PA +) or not detected (PA −) in the associated soil of the sampled tree. Only phyla represented by at least 100 total ASVs are shown. ‘Unidentified fungi’ consists of fungal ASVs not identified to the phylum level

Seven bacterial phyla with at least 100 ASVs were identified across all samples, most of which showed similar trends to that of overall prokaryotes (Fig. 2). Among these, average richness per tree sample was greatest for Proteobacteria, with 2179 (± 167) ASVs identified where *P. agathidicida* was not detected compared to 1778 (± 120) where it was detected (Fig. 2). Acidobacteria, Planctomycetes, and Cyanobacteria had average richness values per tree sample of 439 (± 45.6), 264 (± 21.7), and 246 (± 14.1) ASVs, respectively, where *P. agathidicida* was not detected compared to 398 (± 34.5), 208 (± 20.9), and 212 (± 15.4) ASVs, respectively, where the pathogen was detected. Bacteroidetes, Firmicutes, and Actinobacteria had average richness values per tree sample of 71 (± 5.26), 54 (± 4.35), and 43 (± 5.04) ASVs, respectively, where *P. agathidicida* was not detected compared to 71 (± 4.01), 47 (± 3.61), and 27 (± 2.82) ASVs respectively where the pathogen was detected. Statistical support for these richness differences was detected for Proteobacteria (*t*_44.4_ = 3.053, *p* = 0.004) and Actinobacteria (*t*_26.3_ = 3.369, *p* = 0.002).

Ascomycota and Basidiomycota were the only fungal phyla detected with at least 100 ASVs (Fig. 2). Across the three sites, the average richness per tree sample of Ascomycota was 50 (± 4.66) ASVs where *P. agathidicida* was not detected and 44 (± 3.24) where it was detected. The average richness per tree sample of Basidiomycota was 25 (± 1.82) ASVs where *P. agathidicida* was not detected and 20 (± 1.22) where it was detected. Similarly, the mean richness of ‘unidentified fungi’ was 24 (± 2.66) ASVs where *P. agathidicida* was not detected and 21.4 (± 2.0) where it was detected. Statistical support for fungal richness differences was detected only for Basidiomycota (*t*_28.3_ = 2.442, *p* = 0.021).

**Fig. 3:**
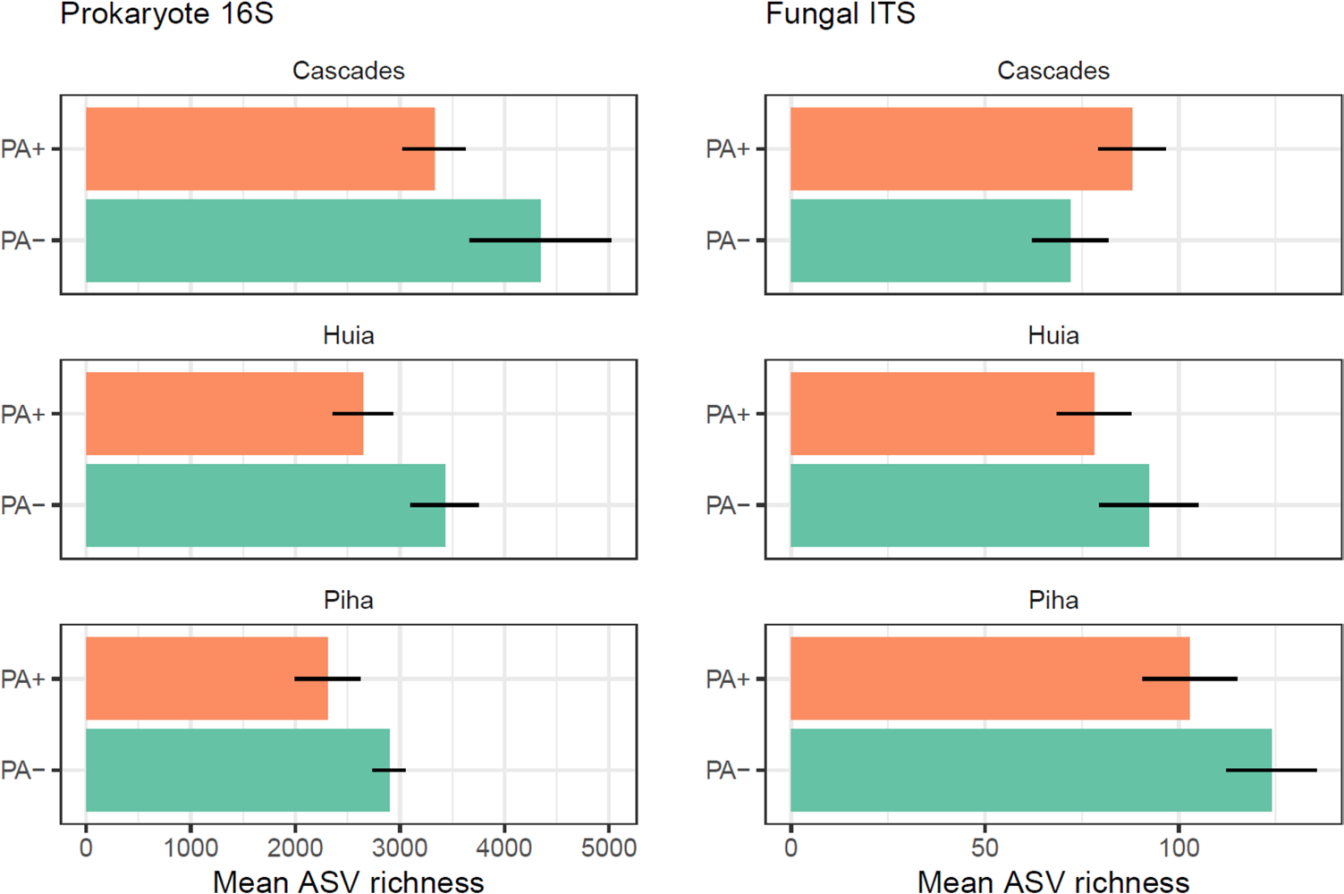
Mean ASV richness (± SE) of prokaryotes and fungi on the phyllosphere (i.e. leaf surface) of *A. australis*. Comparisons are between conditions where *P. agathidicida* was detected (PA +) or not detected (PA −) in the associated soil at three different study sites (Cascades, Huia, and Piha). Error bars represent standard error. There were no significant differences found between PA + and PA − by site (*p* > 0.05)

Patterns of lower average richness of prokaryote and fungal ASVs where the pathogen was detected were also observed at most individual sites (Fig. 3). The largest richness difference was observed for prokaryotes at Cascades, with 4344 (± 677) ASVs where *P. agathidicida* was not detected compared to 3327 (± 302) where it was detected. None of these site-level differences was supported by statistical evidence however (*p* > 0.05).

### Leaf Leachates and Phyllosphere Community Structure

Leaf leachate nutrient composition and pH varied across sites and between *P. agathidicida* detected and not detected conditions (Table 1; Supplemental Fig. 2a–b). The ranges for each elemental concentration (ppm) for trees under each condition largely overlapped, with some exceptions. For example, for Cascades, negligible B and Si were detected, while Piha and Huia sites had much higher ranges. Piha also presented higher Ca, Mg, Mn, Na, and Zn concentrations. Despite these site-level differences, it should be noted that leaf leachate pH ranges for each site overlapped under both *P. agathidicida* conditions.

**Table 1:** Nutrients and pH in *Agathis australis* leaf leachates by pathogen detection condition (PA + , *Phytophthora agathidicida* detected; PA − , *Phytophthora agathidicida* not detected) and site. Range (minimum–maximum) of element concentration (ppm) and pH

Element/pH		Unit	Cascades		Huia		Piha	
			PA +	PA −	PA +	PA −	PA +	PA −
Boron	B	ppm	< 0.001–0.02	< 0.001	0.25–0.51	0.29–0.44	0.20–0.21	0.22–0.28
Calcium	Ca	ppm	0.19–0.52	0.26–0.42	0.30–0.78	0.19–0.68	0.81–1.63	1.09–1.50
Carbon	C	ppm	0.30–5.58	2.03–3.35	2.71–9.92	0.92–3.81	2.90–3.64	2.47–5.84
Magnesium	Mg	ppm	0.15–0.46	0.17–0.49	0.37–0.78	0.19–0.78	1.33–2.83	1.44–2.31
Manganese	Mn	ppm	0.01–0.04	0.01–0.02	0.01–0.03	0.01–0.05	0.04–0.08	0.04–0.13
Nitrogen	N	ppm	0.02–0.18	0.05–0.12	0.04–0.14	0.04–0.08	0.02–0.12	0.03–0.09
Potassium	K	ppm	0.27–0.79	0.51–0.73	0.73–1.14	0.43–1.18	0.89–1.29	0.80–1.02
Silicon	Si	ppm	< 0.001	< 0.001	11.16–21.31	14.74–24.15	29.61–31.67	30.27–31.79
Sodium	Na	ppm	0.08–1.49	0.31–1.84	1.28–2.39	0.78–2.28	5.65–11.70	3.14–8.11
Zinc	Zn	ppm	0.01–0.12	0.01–0.09	0.02–0.09	0.01–0.07	0.07–0.21	0.08–0.33
pH			5.08–5.51	5.08–5.38	5.29–5.43	5.30–5.44	5.07–5.31	5.12–4.20

**Fig. 4:**
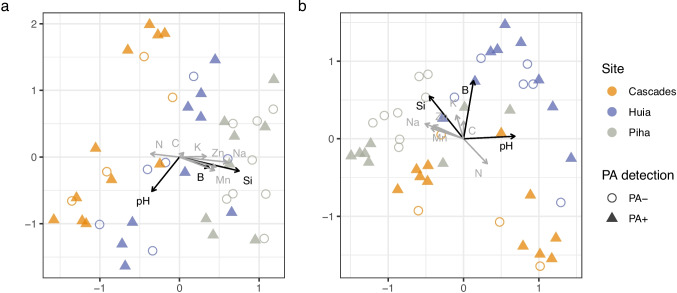
Ordinations of prokaryote (**a**) and fungal (**b**) community structure, based on Bray–Curtis distances between proportional ASV abundances per sample (total = 48 (2 × 24) samples), overlaid with vectors indicating the direction and magnitude of linear trends between leaf leachate variables and community structure. Significant trends (*p* < 0.05) are black, and all others grey. *Phytophthora agathidicida* was detected (PA +) or not detected (PA −) in the associated soil

Ordinations of multivariate community structure indicated that the composition of prokaryote and fungal phyllosphere communities tended to differ between the three sites more clearly than according to *P. agathidicida* detection condition (Fig. 4). Leaf-to-leaf and within-tree variations were not able to be tested. Testing for community differences between sites and between *P. agathidicida* detection conditions using PERMANOVA found evidence of differing 16S and ITS communities between sites (*p* ≥ 0.002), but not pathogen detection condition. Distance-based redundancy analysis indicated that these community differences were most strongly related to varying levels of leaf leachate pH, followed by B and Si concentrations (*F*_1_ = 1.87 to 2.73, *p* = 0.039 to 0.003). Leaf leachate pH was associated with differing prokaryote community composition among samples within each of the sites. Prokaryote communities from Piha tended to be positively associated with B and Si concentrations, whereas the opposite was true of prokaryote communities from Cascades (Fig. 4a). Fungal communities from Piha tended to be positively associated with Si concentration and negatively associated with pH, while fungal communities from Huia tended to be positively associated with B concentration and/or pH. Fungal communities from Cascades tended to be negatively associated with leaf leachate B concentrations; some appear to be associated with low Si concentration and high pH and others with moderate Si concentrations and low pH (Fig. 4b).

## Discussion

The bacterial component of the phyllosphere (i.e. leaf surface) of *Agathis australis* was dominated by Proteobacteria and Acidobacteria, which have been found to be core components of phyllosphere communities in other systems [39, 40]. The ubiquity of Proteobacteria across all samples in this study may be a reflection of the abundance of Proteobacteria in the source communities, the suitability of the leaf surface (e.g. an acidic environment) of *A. australis* to this phylum, and/or the adaptive and competitive advantages this group of bacteria possesses over other groups of bacteria colonising, or attempting to colonise, the *A. australis* leaf surface [41]. The fungal portion of the phyllosphere was dominated by Ascomycota and Basidiomycota, which also are the dominant fungal phyla in plant microbiomes, with most species in these groups associated with plants either as saprobes, parasites, pathogens, or symbionts [42]. Given that only 23 unique Ascomycota species from *A. australis* leaves of various conditions have previously been recorded [23], this study’s finding of more than 40 Ascomycota ASVs from fresh leaves only is likely indicative of the advances of culture-independent methods (such as the ITS amplification in this study) that enables a greater diversity of fungi to be detected from the *A. australis* leaf surface than culture-dependent methods that selects for fast-growing fungi (e.g. [43]). Furthermore, while Basidiomycota have been noted on the phylloplane of other tree species (e.g. [44, 45]), this study’s identification of Basidiomycota on the fresh leaves of *A. australis* is a first. Many species of Ascomycota and Basidiomycota have been identified as important phyllosphere inhabitants of multiple plant species [46], and certain species have been known to provide protection against pathogens [47].

Overall, our study demonstrated that the presence of *Phytophthora agathidicida* in soils affected the phyllosphere community of *A. australis*. The dominant Proteobacteria was recovered significantly more often in the phyllosphere of trees that were associated with soil in which *P. agathidicida* had not been detected, which is similar to a previous study of the impact of *P. agathidicida* on soil microbiota [16]. However, the second most abundant phylum, Acidobacteria, was found in higher relative abundance in the phyllosphere of trees that were associated with soils in which *P. agathidicida* had not been detected, which contrasts with a previous study in which Acidobacteria was more abundant in soils in which *P. agathidicida* had been detected [16]. We did not find an overall shift in phyllosphere fungal community composition between trees where *P. agathidicida* was detected and not detected in the associated soil. In contrast, higher soil fungal diversity of symptomatic *A. australis* soils compared to asymptomatic *A. australis* soils was reported by Byers et al. [16]. This observation was explained by secondary colonisation processes, in particular by saprophytic fungi following tree death. The relative abundance in Ascomycota or Basidiomycota tended to be lower in trees where *P. agathidicida* was detected in the soil, but differences were not significant.

Differences in bacterial and fungal phyla between trees where *P. agathidicida* was detected and not detected in the soil are likely due to modifications in soil and plant characteristics following *P. agathidicida* infection. Lower forest floor depth [27] and body mass of omnivorous soil mesofauna [48] at locations where *P. agathidicida* was detected are likely resulting in a gradual change of ecosystem processes (e.g. decomposition) and soil nutrient availability. Lower fine root biomass (unpublished data) at locations where *P. agathidicida* was detected impacts nutrient transport through the tree [14] and may partly explain lower leaf phosphorus and potassium concentrations [49] and a decline of canopy and forest floor carbon and nutrient fluxes with increasing soil *P. agathidicida* DNA concentration [17, 18].

Changes in richness level could impact nutrient cycling and tree health. For example, since Proteobacteria are ecologically important microbes, with several species known to be involved in carbon and nitrogen cycling [50], lowered levels of richness may have knock-on effects for soil health and communities. From a tree health perspective, epiphytic fungi have been known to induce systemic resistance to disease in some plant species (e.g. [51]) and Actinobacteria are known to facilitate overall plant health [52]. Lower microbial richness where *P. agathidicida* was detected may therefore raise the question of possible increased risk of secondary disease or pest instances on *A. australis* affected by the pathogen.

Boron and Si were the only leaf leachate nutrients significantly associated with bacterial and fungal phyllosphere community composition. Both Si and B have been shown to mitigate abiotic and biotic stresses in plants through the enhancement of the antioxidant defense system (e.g. [53]). However, preliminary explorations of the data imply that this association may be driven by the nearly nil amounts of B and Si detected at the Cascades site. Even so, this site cannot be treated as an outlier. Instead, the unique characteristics of the Cascades site (the trees were much taller at this site, and the average richness of fungal ASVs where the pathogen was detected was higher than where it was not detected) as well as the non-overlapping ranges for other leachate chemicals between other sites regardless of pathogen detection condition serve as a reminder that site-level differences are important to consider when teasing out the role diseases play in natural ecosystems. Microbial communities have been noted to differ between forest types [45], on the same host plant along ecological gradients (e.g. [54]), and leaf structure and physiology [40]. Future research should consider increasing the number of replicates at a site level to improve the statistical robustness of site-level differences and clarify the role site conditions play on *A. australis* microbial communities.

As opposed to leaf leachate nutrient concentrations, leaf leachate pH varied by individual tree but with no discernable difference between sites. Further, pH was associated with differing prokaryote community composition among samples within each of the sites. This implies that pH is a significant driver of prokaryote and fungal community structure in the *A. australis* phyllosphere. It is well established that pH is a highly important factor influencing rhizosphere and soil microbial communities [55, 56]. Similar to results on bacterial communities in soil environments, bacterial leaf surface communities increase with increasing leaf surface pH [57]. This has been explained by distinct bacterial lineages having distinct conservation of microbial pH preference and a strong effect of pH on microbial metabolism [58].

Our findings suggest that both prokaryote (bacterial) and fungal phyla are less abundant on the leaf surface component of the *A. australis* phyllosphere where *P. agathidicida* was detected in the soil than where it was not detected. This study expands the collective knowledge of *A. australis* microbial communities generally, adds to the body of literature on the effect of *P. agathidicida* on soil and plant characteristics, and contributes to an understanding of broader changes that have or may yet be observed in *A. australis* forest ecosystems.

## Supplementary Information

Below is the link to the electronic supplementary material.

Supplementary file1 (PNG 8114 KB)

Supplementary file2 (PNG 33 KB)

Supplementary file3 (PNG 28 KB)

Supplementary file4 (DOCX 15 KB)

## Data Availability

The data in this research were collected from the traditional lands of Te Kawerau ā Maki with their permission. The authors recognise their sovereignty and authority to control data about their lands under the CARE Principles for Indigenous Data Governance. Requests to access the data should be directed to l.schwendenmann@auckland.ac.nz.
