# Supplementary material for: Phyllosphere of Agathis australis Leaves and the Impact of the Soil-Borne Pathogen Phytophthora agathidicida
Source: Microb Ecol. 2024 Oct 9;87:125. doi: 10.1007/s00248-024-02441-9 (PMC11481638; doi:10.1007/s00248-024-02441-9)
Supplement: Supplementary file 1 [file 248_2024_2441_MOESM4_ESM.docx]

Supplemental Table 1: Summary of the visual canopy health scores and detection of *P. agathidicida* of sampled trees*.* C=Cascades, P=Piha, H=Huia; A=asymptomatic, S=symptomatic.

**Kauri	*P. agathidicida* detection	Canopy Visual Health	(y/n)	Score (1-5)**

CA1	Y	3.0

CA2	Y	2.5

CA3	N	2.5

CA4	Y	2.0

CS1	Y	2.5

CS2	Y	3.0

CS3	N	2.5

CS4	Y	2.5

PA1	N	2.0

PA2	N	2.5

PA3	N	2.5

PA4	N	3.0

PS1	Y	3.0

PS2	Y	2.0

PS3	Y	3.0

PS4	Y	3.5

HA1	Y	2.0

HA2	Y	1.5

HA3	N	1.5

HA4	N	2.5

HS1	Y	3.5

HS2	Y	2.0

HS3	Y	2.0

HS4	N	3.5
