# Supplementary figures and images for: Phyllosphere of Agathis australis Leaves and the Impact of the Soil-Borne Pathogen Phytophthora agathidicida
Source: Microb Ecol. 2024 Oct 9;87(1):125. doi: 10.1007/s00248-024-02441-9 (PMC11481638; doi:10.1007/s00248-024-02441-9)

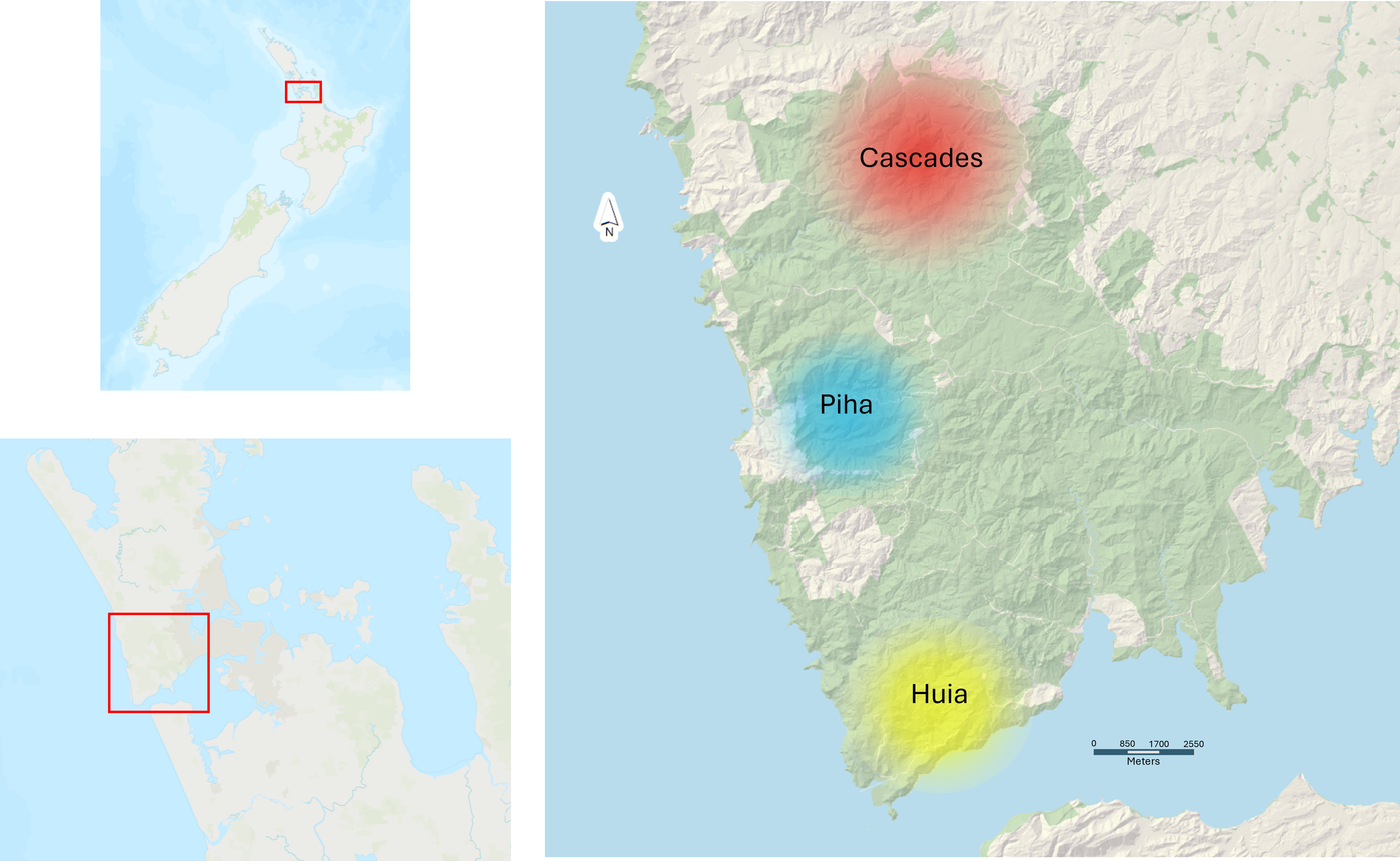

Supplement: Supplementary file 1 — Supplementary file1 (PNG 8114 KB) [file 248_2024_2441_MOESM1_ESM.png]

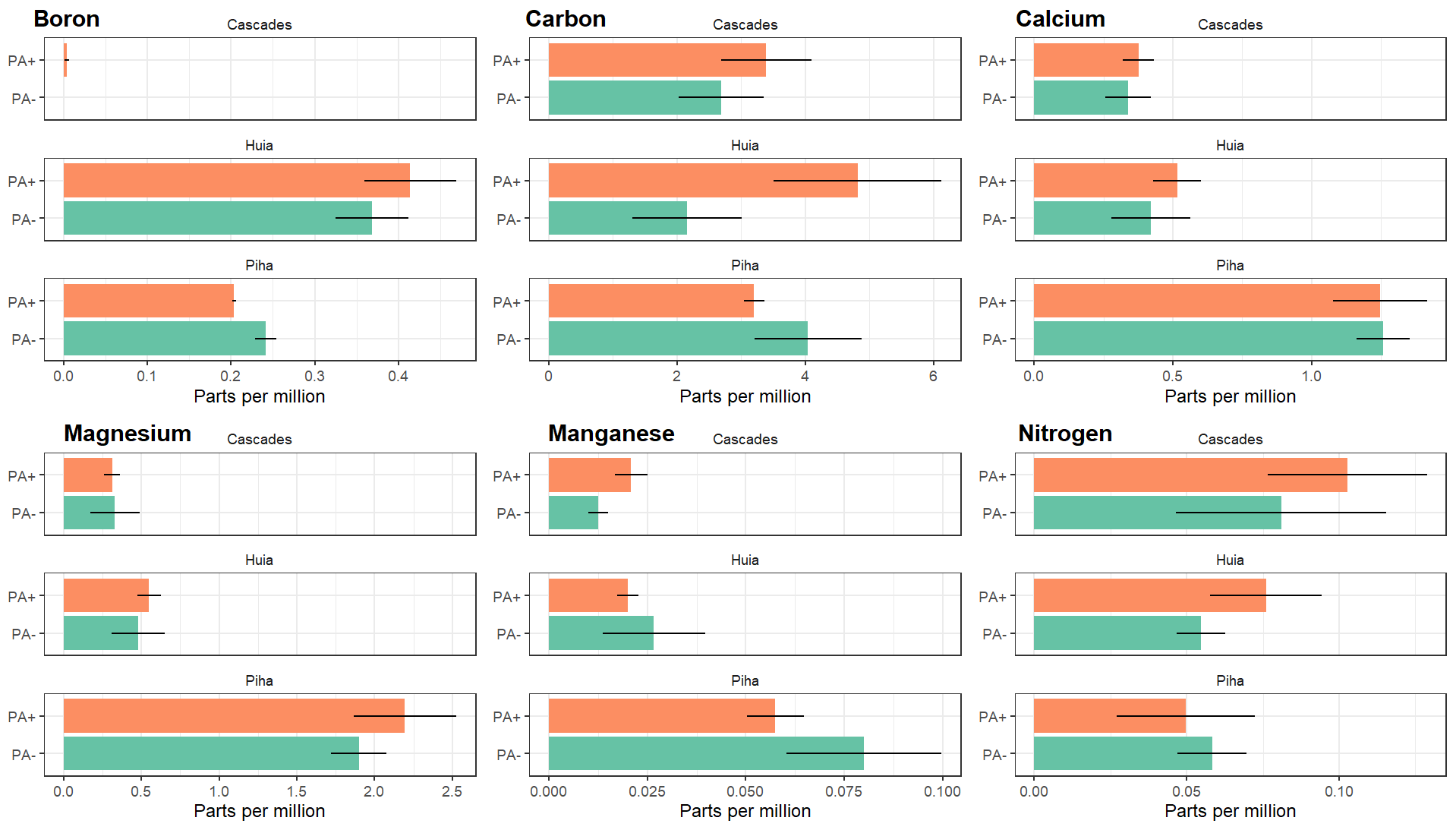

Supplement: Supplementary file 2 — Supplementary file2 (PNG 33 KB) [file 248_2024_2441_MOESM2_ESM.png]

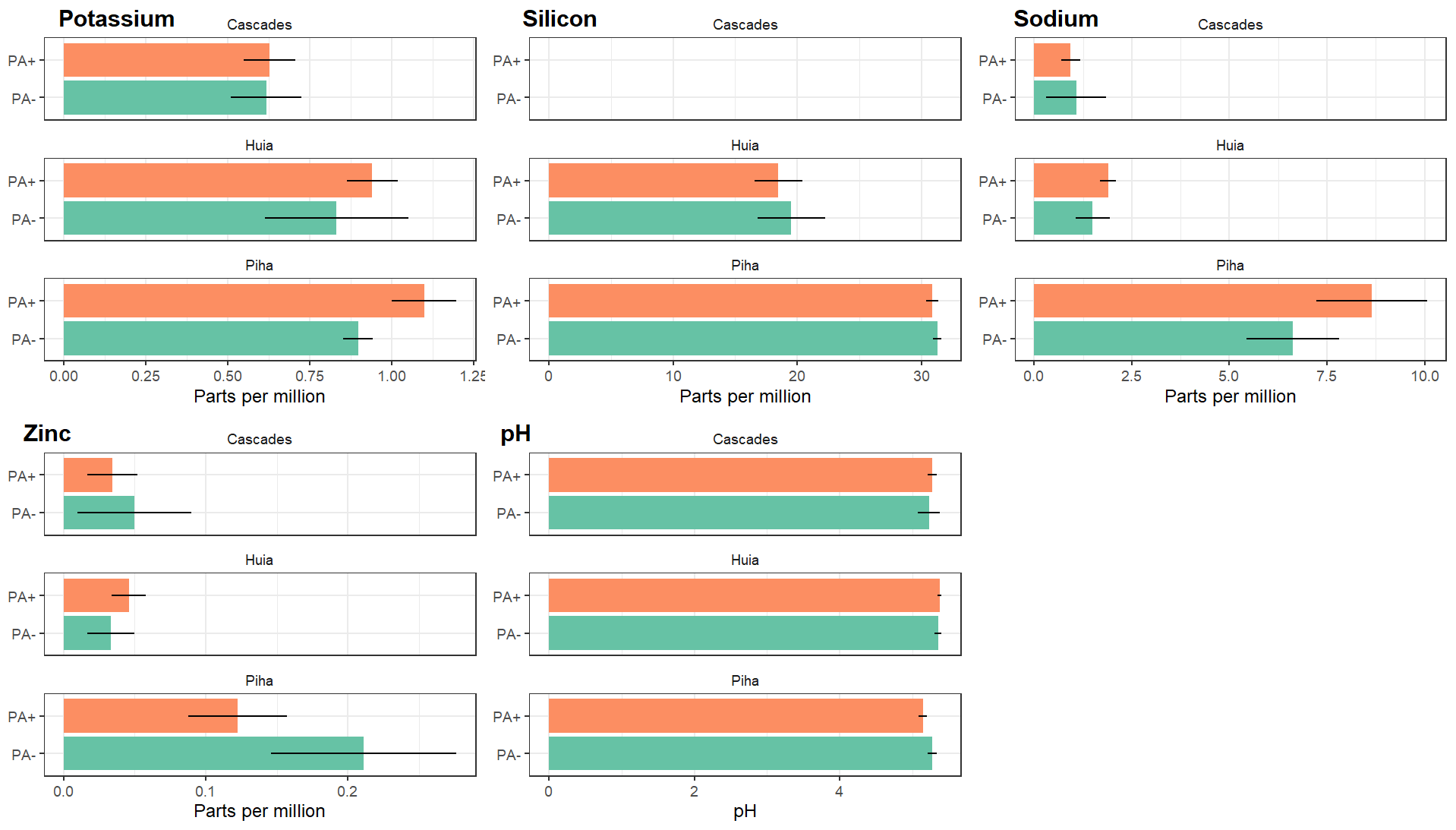

Supplement: Supplementary file 3 — Supplementary file3 (PNG 28 KB) [file 248_2024_2441_MOESM3_ESM.png]
