# Supplementary material for: Phyllosphere of Agathis australis Leaves and the Impact of the Soil-Borne Pathogen Phytophthora agathidicida
Source: Microb Ecol. 2024 Oct 9;87(1):125. doi: 10.1007/s00248-024-02441-9 (PMC11481638; doi:10.1007/s00248-024-02441-9)
Supplement: Supplementary file 4 — Supplementary file4 (DOCX 15 KB) [file 248_2024_2441_MOESM4_ESM.docx]

Supplemental Table 1: Summary of the visual canopy health scores and detection of *P. agathidicida* of sampled trees*.* C=Cascades, P=Piha, H=Huia; A=asymptomatic, S=symptomatic.

**Kauri *P. agathidicida* detection Canopy Visual Health (y/n) Score (1-5)**

CA1 Y 3.0

CA2 Y 2.5

CA3 N 2.5

CA4 Y 2.0

CS1 Y 2.5

CS2 Y 3.0

CS3 N 2.5

CS4 Y 2.5

PA1 N 2.0

PA2 N 2.5

PA3 N 2.5

PA4 N 3.0

PS1 Y 3.0

PS2 Y 2.0

PS3 Y 3.0

PS4 Y 3.5

HA1 Y 2.0

HA2 Y 1.5

HA3 N 1.5

HA4 N 2.5

HS1 Y 3.5

HS2 Y 2.0

HS3 Y 2.0

HS4 N 3.5
